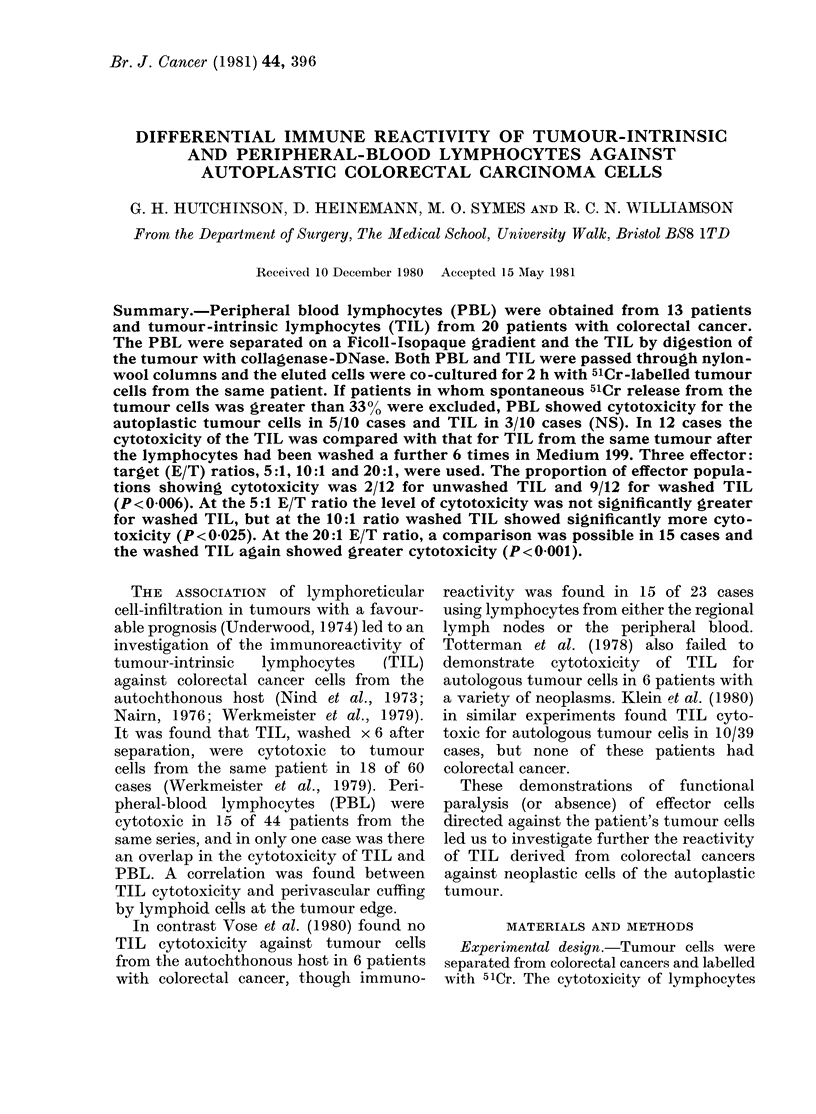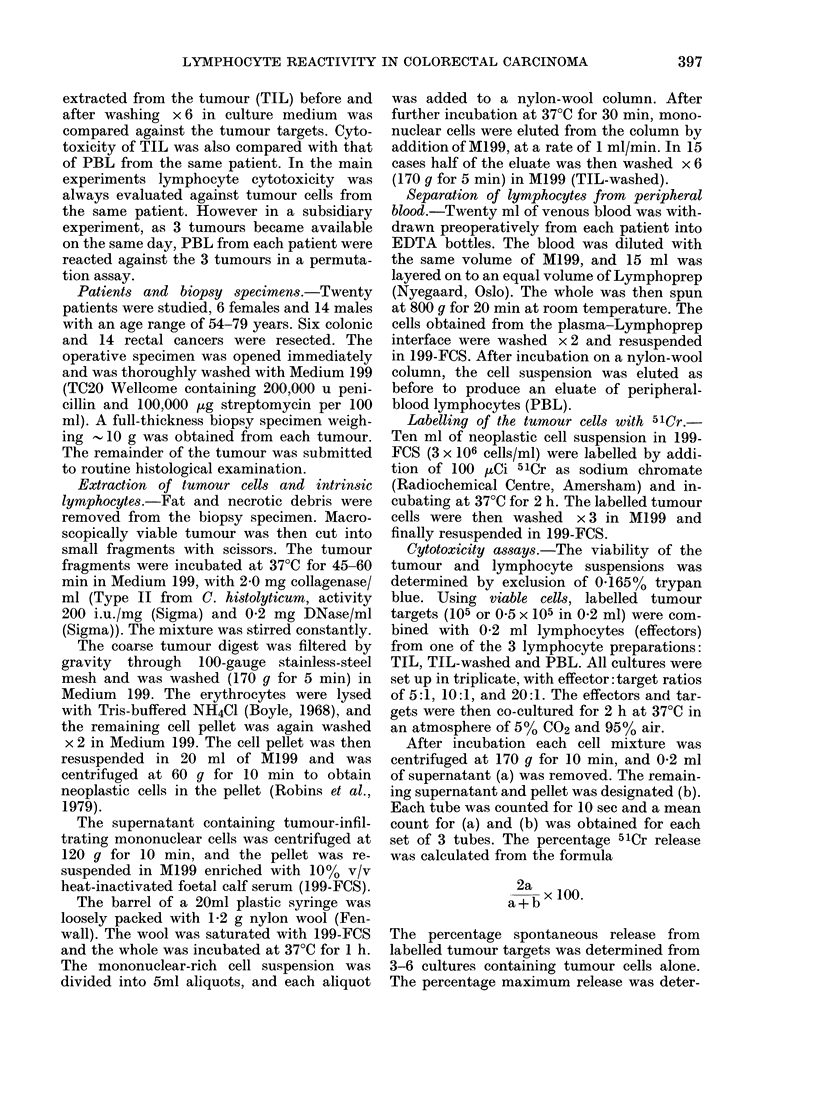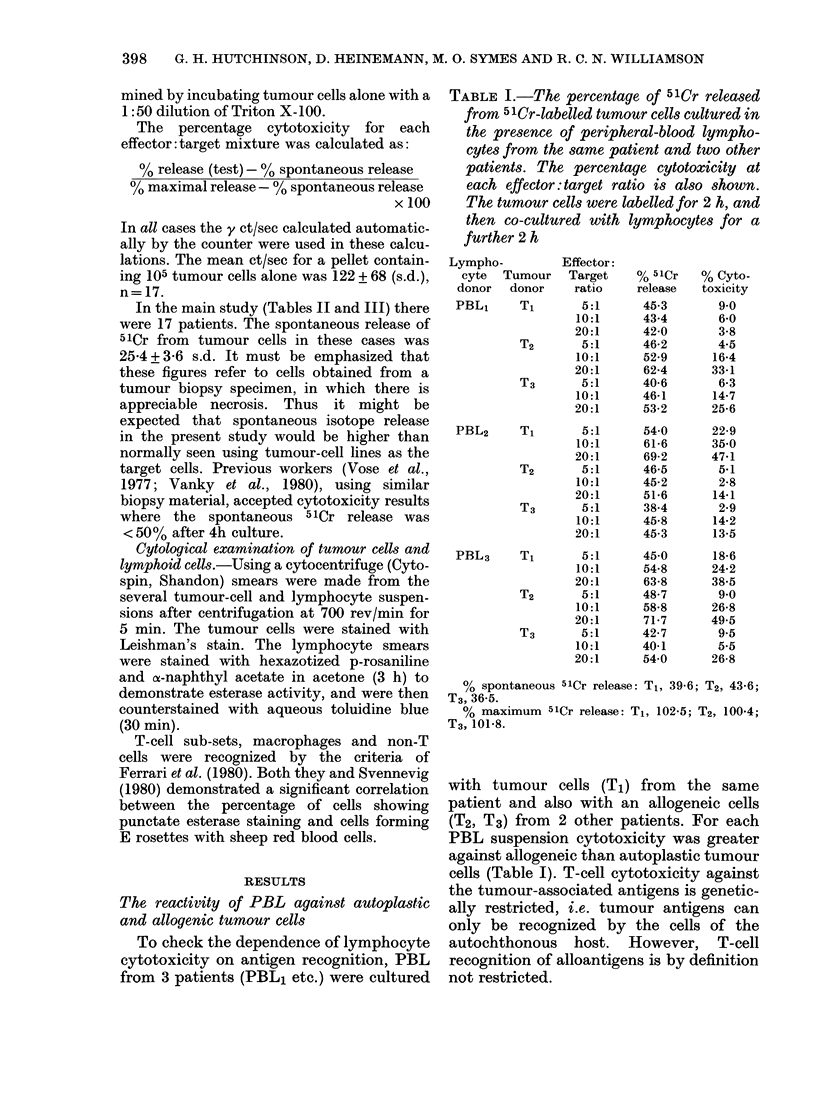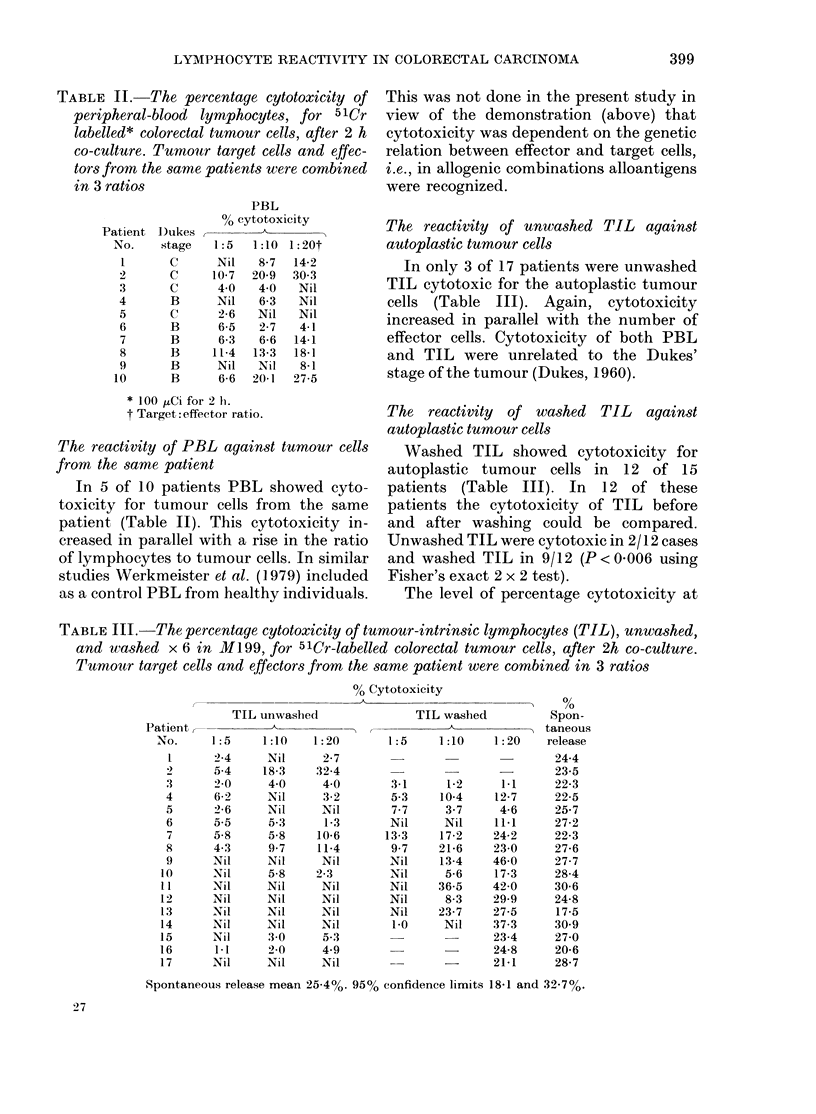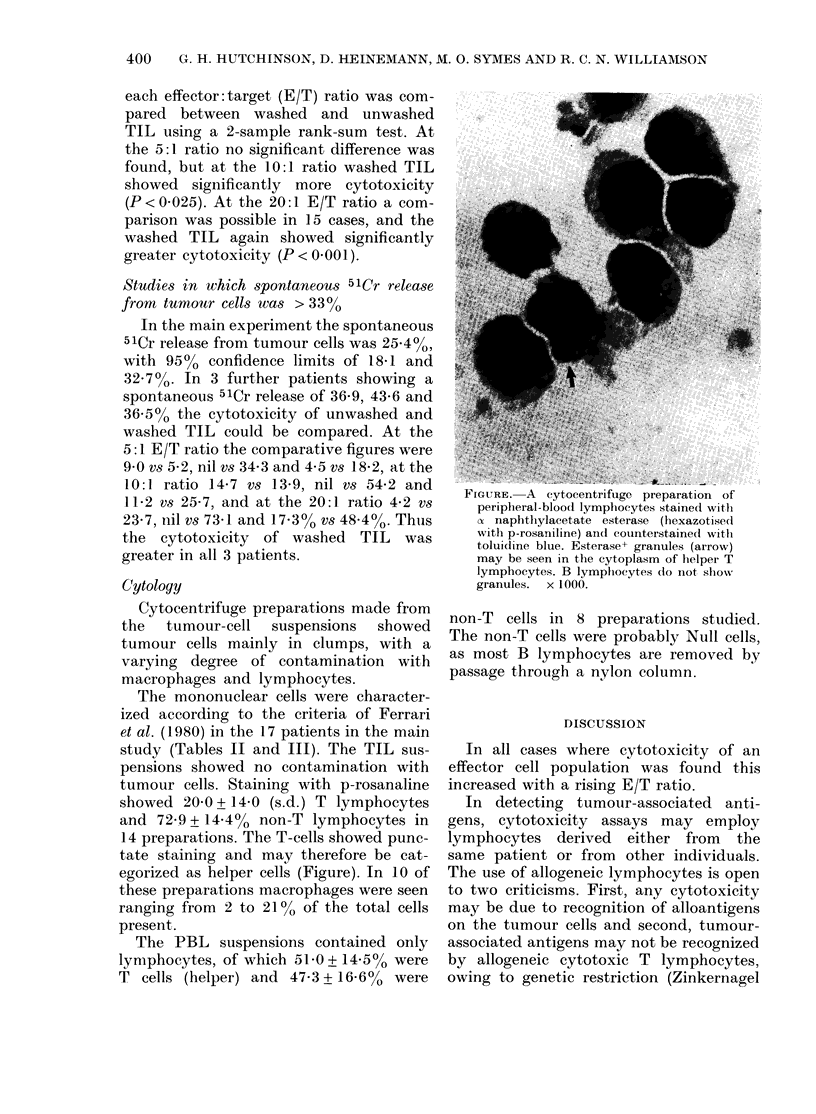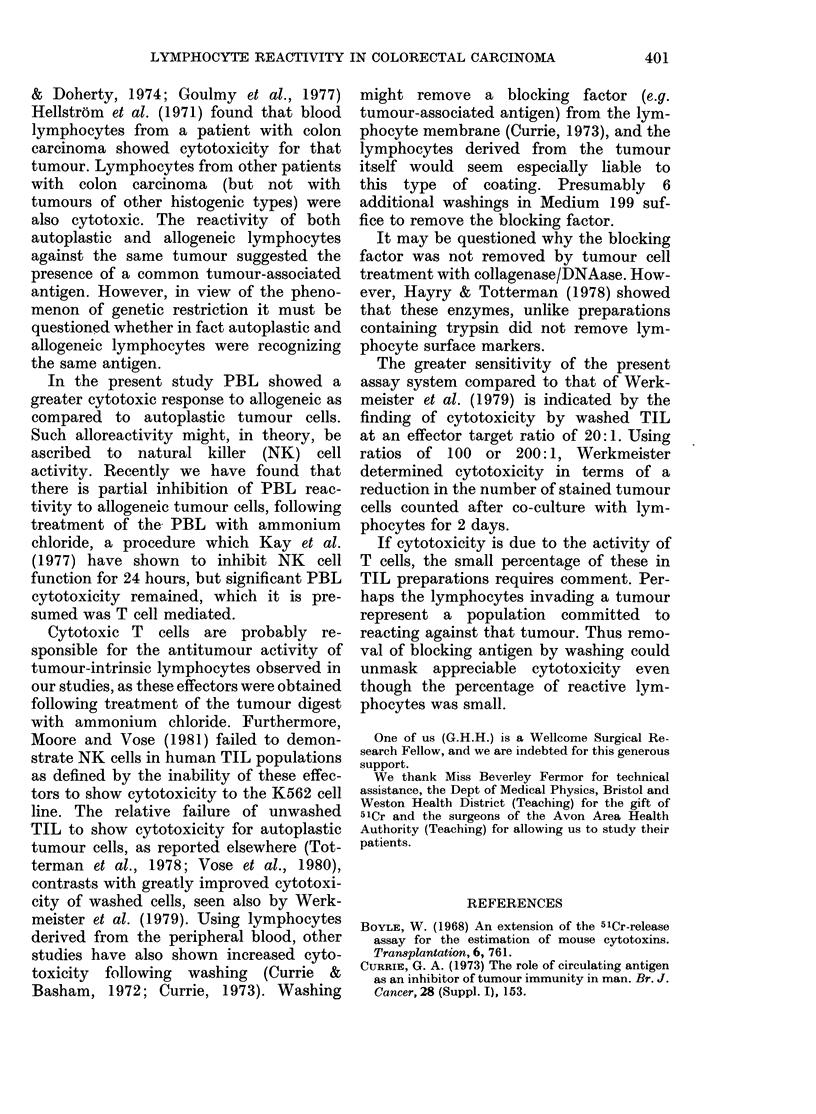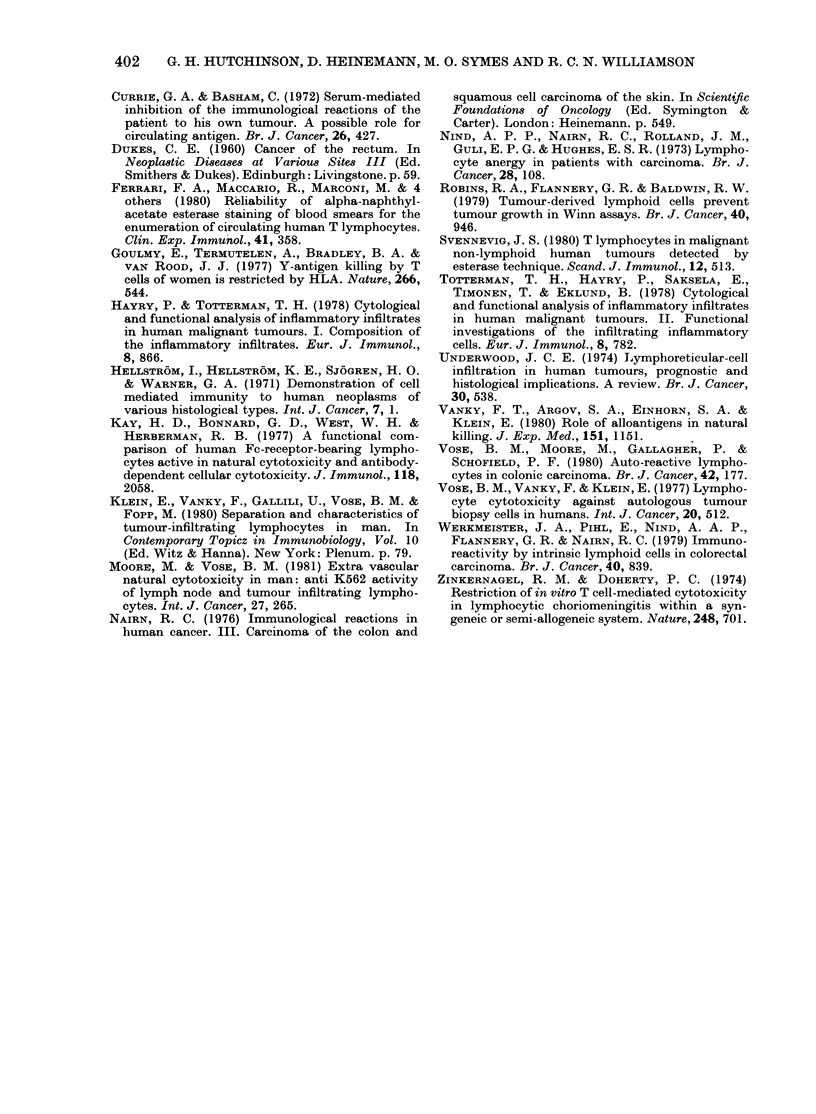# Differential immune reactivity of tumour-intrinsic and peripheral-blood lymphocytes against autoplastic colorectal carcinoma cells.

**DOI:** 10.1038/bjc.1981.197

**Published:** 1981-09

**Authors:** G. H. Hutchinson, D. Heinemann, M. O. Symes, R. C. Williamson

## Abstract

**Images:**


*Br. J. Cancer* (1981) **44**, 396

### DIFFERENTIAL IMMUNE REACTIVITY OF TUMOUR-INTRINSIC AND PERIPHERAL-BLOOD LYMPHOCYTES AGAINST AUTOPLASTIC COLORECTAL CARCINOMA CELLS

G. H. HUTCHINSON, D. HEINEMANN, M. O. SYMES AND R. C. N. WILLIAMSON

*From the Department of Surgery, The Medical School, University Walk, Bristol BS8 1TD*

Received 10 December 1980 Accepted 15 May 1981

**Summary.—Peripheral blood lymphocytes (PBL) were obtained from 13 patients and tumour-intrinsic lymphocytes (TIL) from 20 patients with colorectal cancer. The PBL were separated on a Ficoll-Isopaque gradient and the TIL by digestion of the tumour with collagenase-DNase. Both PBL and TIL were passed through nylon-wool columns and the eluted cells were co-cultured for 2 h with $^{51}$Cr-labelled tumour cells from the same patient. If patients in whom spontaneous $^{51}$Cr release from the tumour cells was greater than 33% were excluded, PBL showed cytotoxicity for the autoplastic tumour cells in 5/10 cases and TIL in 3/10 cases (NS). In 12 cases the cytotoxicity of the TIL was compared with that for TIL from the same tumour after the lymphocytes had been washed a further 6 times in Medium 199. Three effector: target (E/T) ratios, 5:1, 10:1 and 20:1, were used. The proportion of effector populations showing cytotoxicity was 2/12 for unwashed TIL and 9/12 for washed TIL ($P<0{\cdot}006$). At the 5:1 E/T ratio the level of cytotoxicity was not significantly greater for washed TIL, but at the 10:1 ratio washed TIL showed significantly more cytotoxicity ($P<0{\cdot}025$). At the 20:1 E/T ratio, a comparison was possible in 15 cases and the washed TIL again showed greater cytotoxicity ($P<0{\cdot}001$).**

THE ASSOCIATION of lymphoreticular cell-infiltration in tumours with a favourable prognosis (Underwood, 1974) led to an investigation of the immunoreactivity of tumour-intrinsic lymphocytes (TIL) against colorectal cancer cells from the autochthonous host (Nind *et al.*, 1973; Nairn, 1976; Werkmeister *et al.*, 1979). It was found that TIL, washed ×6 after separation, were cytotoxic to tumour cells from the same patient in 18 of 60 cases (Werkmeister *et al.*, 1979). Peripheral-blood lymphocytes (PBL) were cytotoxic in 15 of 44 patients from the same series, and in only one case was there an overlap in the cytotoxicity of TIL and PBL. A correlation was found between TIL cytotoxicity and perivascular cuffing by lymphoid cells at the tumour edge.

In contrast Vose *et al.* (1980) found no TIL cytotoxicity against tumour cells from the autochthonous host in 6 patients with colorectal cancer, though immunoreactivity was found in 15 of 23 cases using lymphocytes from either the regional lymph nodes or the peripheral blood. Totterman *et al.* (1978) also failed to demonstrate cytotoxicity of TIL for autologous tumour cells in 6 patients with a variety of neoplasms. Klein *et al.* (1980) in similar experiments found TIL cytotoxic for autologous tumour cells in 10/39 cases, but none of these patients had colorectal cancer.

These demonstrations of functional paralysis (or absence) of effector cells directed against the patient's tumour cells led us to investigate further the reactivity of TIL derived from colorectal cancers against neoplastic cells of the autoplastic tumour.

### MATERIALS AND METHODS

*Experimental design.*—Tumour cells were separated from colorectal cancers and labelled with $^{51}$Cr. The cytotoxicity of lymphocytes extracted from the tumour (TIL) before and after washing ×6 in culture medium was compared against the tumour targets. Cytotoxicity of TIL was also compared with that of PBL from the same patient. In the main experiments lymphocyte cytotoxicity was always evaluated against tumour cells from the same patient. However in a subsidiary experiment, as 3 tumours became available on the same day, PBL from each patient were reacted against the 3 tumours in a permutation assay.

*Patients and biopsy specimens.*—Twenty patients were studied, 6 females and 14 males with an age range of 54–79 years. Six colonic and 14 rectal cancers were resected. The operative specimen was opened immediately and was thoroughly washed with Medium 199 (TC20 Wellcome containing 200,000 u penicillin and 100,000 μg streptomycin per 100 ml). A full-thickness biopsy specimen weighing ~10 g was obtained from each tumour. The remainder of the tumour was submitted to routine histological examination.

*Extraction of tumour cells and intrinsic lymphocytes.*—Fat and necrotic debris were removed from the biopsy specimen. Macroscopically viable tumour was then cut into small fragments with scissors. The tumour fragments were incubated at 37°C for 45–60 min in Medium 199, with 2·0 mg collagenase/ml (Type II from *C. histolyticum*, activity 200 i.u./mg (Sigma) and 0·2 mg DNase/ml (Sigma)). The mixture was stirred constantly.

The coarse tumour digest was filtered by gravity through 100-gauge stainless-steel mesh and was washed (170 *g* for 5 min) in Medium 199. The erythrocytes were lysed with Tris-buffered $NH_4Cl$ (Boyle, 1968), and the remaining cell pellet was again washed ×2 in Medium 199. The cell pellet was then resuspended in 20 ml of M199 and was centrifuged at 60 *g* for 10 min to obtain neoplastic cells in the pellet (Robins *et al.*, 1979).

The supernatant containing tumour-infiltrating mononuclear cells was centrifuged at 120 *g* for 10 min, and the pellet was resuspended in M199 enriched with 10% v/v heat-inactivated foetal calf serum (199-FCS).

The barrel of a 20ml plastic syringe was loosely packed with 1·2 g nylon wool (Fenwall). The wool was saturated with 199-FCS and the whole was incubated at 37°C for 1 h. The mononuclear-rich cell suspension was divided into 5ml aliquots, and each aliquot was added to a nylon-wool column. After further incubation at 37°C for 30 min, mononuclear cells were eluted from the column by addition of M199, at a rate of 1 ml/min. In 15 cases half of the eluate was then washed ×6 (170 *g* for 5 min) in M199 (TIL-washed).

*Separation of lymphocytes from peripheral blood.*—Twenty ml of venous blood was withdrawn preoperatively from each patient into EDTA bottles. The blood was diluted with the same volume of M199, and 15 ml was layered on to an equal volume of Lymphoprep (Nyegaard, Oslo). The whole was then spun at 800 *g* for 20 min at room temperature. The cells obtained from the plasma–Lymphoprep interface were washed ×2 and resuspended in 199-FCS. After incubation on a nylon-wool column, the cell suspension was eluted as before to produce an eluate of peripheral-blood lymphocytes (PBL).

*Labelling of the tumour cells with $^{51}Cr$.*—Ten ml of neoplastic cell suspension in 199-FCS ($3 \times 10^6$ cells/ml) were labelled by addition of 100 μCi $^{51}$Cr as sodium chromate (Radiochemical Centre, Amersham) and incubating at 37°C for 2 h. The labelled tumour cells were then washed ×3 in M199 and finally resuspended in 199-FCS.

*Cytotoxicity assays.*—The viability of the tumour and lymphocyte suspensions was determined by exclusion of 0·165% trypan blue. Using *viable cells*, labelled tumour targets ($10^5$ or $0{\cdot}5 \times 10^5$ in 0·2 ml) were combined with 0·2 ml lymphocytes (effectors) from one of the 3 lymphocyte preparations: TIL, TIL-washed and PBL. All cultures were set up in triplicate, with effector:target ratios of 5:1, 10:1, and 20:1. The effectors and targets were then co-cultured for 2 h at 37°C in an atmosphere of 5% $CO_2$ and 95% air.

After incubation each cell mixture was centrifuged at 170 *g* for 10 min, and 0·2 ml of supernatant (a) was removed. The remaining supernatant and pellet was designated (b). Each tube was counted for 10 sec and a mean count for (a) and (b) was obtained for each set of 3 tubes. The percentage $^{51}$Cr release was calculated from the formula

$$\frac{2a}{a+b} \times 100.$$

The percentage spontaneous release from labelled tumour targets was determined from 3–6 cultures containing tumour cells alone. The percentage maximum release was determined by incubating tumour cells alone with a 1:50 dilution of Triton X-100.

The percentage cytotoxicity for each effector:target mixture was calculated as:

$$\frac{\%\ \text{release (test)} - \%\ \text{spontaneous release}}{\%\ \text{maximal release} - \%\ \text{spontaneous release}} \times 100$$

In *all* cases the $\gamma$ ct/sec calculated automatically by the counter were used in these calculations. The mean ct/sec for a pellet containing $10^5$ tumour cells alone was $122 \pm 68$ (s.d.), n = 17.

In the main study (Tables II and III) there were 17 patients. The spontaneous release of $^{51}$Cr from tumour cells in these cases was $25{\cdot}4 \pm 3{\cdot}6$ s.d. It must be emphasized that these figures refer to cells obtained from a tumour biopsy specimen, in which there is appreciable necrosis. Thus it might be expected that spontaneous isotope release in the present study would be higher than normally seen using tumour-cell lines as the target cells. Previous workers (Vose *et al.*, 1977; Vanky *et al.*, 1980), using similar biopsy material, accepted cytotoxicity results where the spontaneous $^{51}$Cr release was <50% after 4h culture.

*Cytological examination of tumour cells and lymphoid cells.*—Using a cytocentrifuge (Cytospin, Shandon) smears were made from the several tumour-cell and lymphocyte suspensions after centrifugation at 700 rev/min for 5 min. The tumour cells were stained with Leishman's stain. The lymphocyte smears were stained with hexazotized p-rosaniline and $\alpha$-naphthyl acetate in acetone (3 h) to demonstrate esterase activity, and were then counterstained with aqueous toluidine blue (30 min).

T-cell sub-sets, macrophages and non-T cells were recognized by the criteria of Ferrari *et al.* (1980). Both they and Svennevig (1980) demonstrated a significant correlation between the percentage of cells showing punctate esterase staining and cells forming E rosettes with sheep red blood cells.

### RESULTS

*The reactivity of PBL against autoplastic and allogenic tumour cells*

To check the dependence of lymphocyte cytotoxicity on antigen recognition, PBL from 3 patients ($PBL_1$ etc.) were cultured with tumour cells ($T_1$) from the same patient and also with an allogeneic cells ($T_2$, $T_3$) from 2 other patients. For each PBL suspension cytotoxicity was greater against allogeneic than autoplastic tumour cells (Table I). T-cell cytotoxicity against the tumour-associated antigens is genetically restricted, *i.e.* tumour antigens can only be recognized by the cells of the autochthonous host. However, T-cell recognition of alloantigens is by definition not restricted.

TABLE I.—*The percentage of $^{51}$Cr released from $^{51}$Cr-labelled tumour cells cultured in the presence of peripheral-blood lymphocytes from the same patient and two other patients. The percentage cytotoxicity at each effector:target ratio is also shown. The tumour cells were labelled for 2 h, and then co-cultured with lymphocytes for a further 2 h*

| Lymphocyte donor | Tumour donor | Effector: Target ratio | % $^{51}$Cr release | % Cytotoxicity |
|---|---|---|---|---|
| $PBL_1$ | $T_1$ | 5:1 | 45·3 | 9·0 |
| | | 10:1 | 43·4 | 6·0 |
| | | 20:1 | 42·0 | 3·8 |
| | $T_2$ | 5:1 | 46·2 | 4·5 |
| | | 10:1 | 52·9 | 16·4 |
| | | 20:1 | 62·4 | 33·1 |
| | $T_3$ | 5:1 | 40·6 | 6·3 |
| | | 10:1 | 46·1 | 14·7 |
| | | 20:1 | 53·2 | 25·6 |
| $PBL_2$ | $T_1$ | 5:1 | 54·0 | 22·9 |
| | | 10:1 | 61·6 | 35·0 |
| | | 20:1 | 69·2 | 47·1 |
| | $T_2$ | 5:1 | 46·5 | 5·1 |
| | | 10:1 | 45·2 | 2·8 |
| | | 20:1 | 51·6 | 14·1 |
| | $T_3$ | 5:1 | 38·4 | 2·9 |
| | | 10:1 | 45·8 | 14·2 |
| | | 20:1 | 45·3 | 13·5 |
| $PBL_3$ | $T_1$ | 5:1 | 45·0 | 18·6 |
| | | 10:1 | 54·8 | 24·2 |
| | | 20:1 | 63·8 | 38·5 |
| | $T_2$ | 5:1 | 48·7 | 9·0 |
| | | 10:1 | 58·8 | 26·8 |
| | | 20:1 | 71·7 | 49·5 |
| | $T_3$ | 5:1 | 42·7 | 9·5 |
| | | 10:1 | 40·1 | 5·5 |
| | | 20:1 | 54·0 | 26·8 |

% spontaneous $^{51}$Cr release: $T_1$, 39·6; $T_2$, 43·6; $T_3$, 36·5.

% maximum $^{51}$Cr release: $T_1$, 102·5; $T_2$, 100·4; $T_3$, 101·8.

TABLE II.—*The percentage cytotoxicity of peripheral-blood lymphocytes, for $^{51}Cr$ labelled* colorectal tumour cells, after 2 h co-culture. Tumour target cells and effectors from the same patients were combined in 3 ratios*

| Patient No. | Dukes stage | PBL % cytotoxicity 1:5 | 1:10 | 1:20† |
|---|---|---|---|---|
| 1 | C | Nil | 8·7 | 14·2 |
| 2 | C | 10·7 | 20·9 | 30·3 |
| 3 | C | 4·0 | 4·0 | Nil |
| 4 | B | Nil | 6·3 | Nil |
| 5 | C | 2·6 | Nil | Nil |
| 6 | B | 6·5 | 2·7 | 4·1 |
| 7 | B | 6·3 | 6·6 | 14·1 |
| 8 | B | 11·4 | 13·3 | 18·1 |
| 9 | B | Nil | Nil | 8·1 |
| 10 | B | 6·6 | 20·1 | 27·5 |

* 100 μCi for 2 h.

† Target:effector ratio.

*The reactivity of PBL against tumour cells from the same patient*

In 5 of 10 patients PBL showed cytotoxicity for tumour cells from the same patient (Table II). This cytotoxicity increased in parallel with a rise in the ratio of lymphocytes to tumour cells. In similar studies Werkmeister *et al.* (1979) included as a control PBL from healthy individuals. This was not done in the present study in view of the demonstration (above) that cytotoxicity was dependent on the genetic relation between effector and target cells, *i.e.*, in allogenic combinations alloantigens were recognized.

*The reactivity of unwashed TIL against autoplastic tumour cells*

In only 3 of 17 patients were unwashed TIL cytotoxic for the autoplastic tumour cells (Table III). Again, cytotoxicity increased in parallel with the number of effector cells. Cytotoxicity of both PBL and TIL were unrelated to the Dukes' stage of the tumour (Dukes, 1960).

*The reactivity of washed TIL against autoplastic tumour cells*

Washed TIL showed cytotoxicity for autoplastic tumour cells in 12 of 15 patients (Table III). In 12 of these patients the cytotoxicity of TIL before and after washing could be compared. Unwashed TIL were cytotoxic in 2/12 cases and washed TIL in 9/12 ($P < 0{\cdot}006$ using Fisher's exact 2 × 2 test).

The level of percentage cytotoxicity at

TABLE III.—*The percentage cytotoxicity of tumour-intrinsic lymphocytes (TIL), unwashed, and washed × 6 in M199, for $^{51}Cr$-labelled colorectal tumour cells, after 2h co-culture. Tumour target cells and effectors from the same patient were combined in 3 ratios*

| Patient No. | % Cytotoxicity: TIL unwashed 1:5 | 1:10 | 1:20 | TIL washed 1:5 | 1:10 | 1:20 | % Spontaneous release |
|---|---|---|---|---|---|---|---|
| 1 | 2·4 | Nil | 2·7 | — | — | — | 24·4 |
| 2 | 5·4 | 18·3 | 32·4 | — | — | — | 23·5 |
| 3 | 2·0 | 4·0 | 4·0 | 3·1 | 1·2 | 1·1 | 22·3 |
| 4 | 6·2 | Nil | 3·2 | 5·3 | 10·4 | 12·7 | 22·5 |
| 5 | 2·6 | Nil | Nil | 7·7 | 3·7 | 4·6 | 25·7 |
| 6 | 5·5 | 5·3 | 1·3 | Nil | Nil | 11·1 | 27·2 |
| 7 | 5·8 | 5·8 | 10·6 | 13·3 | 17·2 | 24·2 | 22·3 |
| 8 | 4·3 | 9·7 | 11·4 | 9·7 | 21·6 | 23·0 | 27·6 |
| 9 | Nil | Nil | Nil | Nil | 13·4 | 46·0 | 27·7 |
| 10 | Nil | 5·8 | 2·3 | Nil | 5·6 | 17·3 | 28·4 |
| 11 | Nil | Nil | Nil | Nil | 36·5 | 42·0 | 30·6 |
| 12 | Nil | Nil | Nil | Nil | 8·3 | 29·9 | 24·8 |
| 13 | Nil | Nil | Nil | Nil | 23·7 | 27·5 | 17·5 |
| 14 | Nil | Nil | Nil | 1·0 | Nil | 37·3 | 30·9 |
| 15 | Nil | 3·0 | 5·3 | — | — | 23·4 | 27·0 |
| 16 | 1·1 | 2·0 | 4·9 | — | — | 24·8 | 20·6 |
| 17 | Nil | Nil | Nil | — | — | 21·1 | 28·7 |

Spontaneous release mean 25·4%. 95% confidence limits 18·1 and 32·7%.

each effector:target (E/T) ratio was compared between washed and unwashed TIL using a 2-sample rank-sum test. At the 5:1 ratio no significant difference was found, but at the 10:1 ratio washed TIL showed significantly more cytotoxicity ($P < 0{\cdot}025$). At the 20:1 E/T ratio a comparison was possible in 15 cases, and the washed TIL again showed significantly greater cytotoxicity ($P < 0{\cdot}001$).

*Studies in which spontaneous $^{51}Cr$ release from tumour cells was > 33%*

In the main experiment the spontaneous $^{51}$Cr release from tumour cells was 25·4%, with 95% confidence limits of 18·1 and 32·7%. In 3 further patients showing a spontaneous $^{51}$Cr release of 36·9, 43·6 and 36·5% the cytotoxicity of unwashed and washed TIL could be compared. At the 5:1 E/T ratio the comparative figures were 9·0 *vs* 5·2, nil *vs* 34·3 and 4·5 *vs* 18·2, at the 10:1 ratio 14·7 *vs* 13·9, nil *vs* 54·2 and 11·2 *vs* 25·7, and at the 20:1 ratio 4·2 *vs* 23·7, nil *vs* 73·1 and 17·3% *vs* 48·4%. Thus the cytotoxicity of washed TIL was greater in all 3 patients.

*Cytology*

Cytocentrifuge preparations made from the tumour-cell suspensions showed tumour cells mainly in clumps, with a varying degree of contamination with macrophages and lymphocytes.

The mononuclear cells were characterized according to the criteria of Ferrari *et al.* (1980) in the 17 patients in the main study (Tables II and III). The TIL suspensions showed no contamination with tumour cells. Staining with p-rosanaline showed 20·0 ± 14·0 (s.d.) T lymphocytes and 72·9 ± 14·4% non-T lymphocytes in 14 preparations. The T-cells showed punctate staining and may therefore be categorized as helper cells (Figure). In 10 of these preparations macrophages were seen ranging from 2 to 21% of the total cells present.

The PBL suspensions contained only lymphocytes, of which 51·0 ± 14·5% were T cells (helper) and 47·3 ± 16·6% were non-T cells in 8 preparations studied. The non-T cells were probably Null cells, as most B lymphocytes are removed by passage through a nylon column.

FIGURE.—A cytocentrifuge preparation of peripheral-blood lymphocytes stained with α naphthylacetate esterase (hexazotised with p-rosaniline) and counterstained with toluidine blue. Esterase+ granules (arrow) may be seen in the cytoplasm of helper T lymphocytes. B lymphocytes do not show granules. ×1000.

DISCUSSION

In all cases where cytotoxicity of an effector cell population was found this increased with a rising E/T ratio.

In detecting tumour-associated antigens, cytotoxicity assays may employ lymphocytes derived either from the same patient or from other individuals. The use of allogeneic lymphocytes is open to two criticisms. First, any cytotoxicity may be due to recognition of alloantigens on the tumour cells and second, tumour-associated antigens may not be recognized by allogeneic cytotoxic T lymphocytes, owing to genetic restriction (Zinkernagel

& Doherty, 1974; Goulmy *et al.*, 1977) Hellström *et al.* (1971) found that blood lymphocytes from a patient with colon carcinoma showed cytotoxicity for that tumour. Lymphocytes from other patients with colon carcinoma (but not with tumours of other histogenic types) were also cytotoxic. The reactivity of both autoplastic and allogeneic lymphocytes against the same tumour suggested the presence of a common tumour-associated antigen. However, in view of the phenomenon of genetic restriction it must be questioned whether in fact autoplastic and allogeneic lymphocytes were recognizing the same antigen.

In the present study PBL showed a greater cytotoxic response to allogeneic as compared to autoplastic tumour cells. Such alloreactivity might, in theory, be ascribed to natural killer (NK) cell activity. Recently we have found that there is partial inhibition of PBL reactivity to allogeneic tumour cells, following treatment of the PBL with ammonium chloride, a procedure which Kay *et al.* (1977) have shown to inhibit NK cell function for 24 hours, but significant PBL cytotoxicity remained, which it is presumed was T cell mediated.

Cytotoxic T cells are probably responsible for the antitumour activity of tumour-intrinsic lymphocytes observed in our studies, as these effectors were obtained following treatment of the tumour digest with ammonium chloride. Furthermore, Moore and Vose (1981) failed to demonstrate NK cells in human TIL populations as defined by the inability of these effectors to show cytotoxicity to the K562 cell line. The relative failure of unwashed TIL to show cytotoxicity for autoplastic tumour cells, as reported elsewhere (Totterman *et al.*, 1978; Vose *et al.*, 1980), contrasts with greatly improved cytotoxicity of washed cells, seen also by Werkmeister *et al.* (1979). Using lymphocytes derived from the peripheral blood, other studies have also shown increased cytotoxicity following washing (Currie & Basham, 1972; Currie, 1973). Washing might remove a blocking factor (*e.g.* tumour-associated antigen) from the lymphocyte membrane (Currie, 1973), and the lymphocytes derived from the tumour itself would seem especially liable to this type of coating. Presumably 6 additional washings in Medium 199 suffice to remove the blocking factor.

It may be questioned why the blocking factor was not removed by tumour cell treatment with collagenase/DNAase. However, Hayry & Totterman (1978) showed that these enzymes, unlike preparations containing trypsin did not remove lymphocyte surface markers.

The greater sensitivity of the present assay system compared to that of Werkmeister *et al.* (1979) is indicated by the finding of cytotoxicity by washed TIL at an effector target ratio of 20:1. Using ratios of 100 or 200:1, Werkmeister determined cytotoxicity in terms of a reduction in the number of stained tumour cells counted after co-culture with lymphocytes for 2 days.

If cytotoxicity is due to the activity of T cells, the small percentage of these in TIL preparations requires comment. Perhaps the lymphocytes invading a tumour represent a population committed to reacting against that tumour. Thus removal of blocking antigen by washing could unmask appreciable cytotoxicity even though the percentage of reactive lymphocytes was small.

One of us (G.H.H.) is a Wellcome Surgical Research Fellow, and we are indebted for this generous support.

We thank Miss Beverley Fermor for technical assistance, the Dept of Medical Physics, Bristol and Weston Health District (Teaching) for the gift of $^{51}Cr$ and the surgeons of the Avon Area Health Authority (Teaching) for allowing us to study their patients.

### REFERENCES

BOYLE, W. (1968) An extension of the $^{51}Cr$-release assay for the estimation of mouse cytotoxins. *Transplantation*, **6**, 761.

CURRIE, G. A. (1973) The role of circulating antigen as an inhibitor of tumour immunity in man. *Br. J. Cancer*, **28** (Suppl. I), 153.

CURRIE, G. A. & BASHAM, C. (1972) Serum-mediated inhibition of the immunological reactions of the patient to his own tumour. A possible role for circulating antigen. *Br. J. Cancer*, **26**, 427.

DUKES, C. E. (1960) Cancer of the rectum. In *Neoplastic Diseases at Various Sites III* (Ed. Smithers & Dukes). Edinburgh: Livingstone. p. 59.

FERRARI, F. A., MACCARIO, R., MARCONI, M. & 4 others (1980) Reliability of alpha-naphthyl-acetate esterase staining of blood smears for the enumeration of circulating human T lymphocytes. *Clin. Exp. Immunol.*, **41**, 358.

GOULMY, E., TERMUTELEN, A., BRADLEY, B. A. & VAN ROOD, J. J. (1977) Y-antigen killing by T cells of women is restricted by HLA. *Nature*, **266**, 544.

HAYRY, P. & TOTTERMAN, T. H. (1978) Cytological and functional analysis of inflammatory infiltrates in human malignant tumours. I. Composition of the inflammatory infiltrates. *Eur. J. Immunol.*, **8**, 866.

HELLSTRÖM, I., HELLSTRÖM, K. E., SJÖGREN, H. O. & WARNER, G. A. (1971) Demonstration of cell mediated immunity to human neoplasms of various histological types. *Int. J. Cancer*, **7**, 1.

KAY, H. D., BONNARD, G. D., WEST, W. H. & HERBERMAN, R. B. (1977) A functional comparison of human Fc-receptor-bearing lymphocytes active in natural cytotoxicity and antibody-dependent cellular cytotoxicity. *J. Immunol.*, **118**, 2058.

KLEIN, E., VANKY, F., GALLILI, U., VOSE, B. M. & FOPP, M. (1980) Separation and characteristics of tumour-infiltrating lymphocytes in man. In *Contemporary Topicz in Immunobiology, Vol.* 10 (Ed. Witz & Hanna). New York: Plenum. p. 79.

MOORE, M. & VOSE, B. M. (1981) Extra vascular natural cytotoxicity in man: anti K562 activity of lymph node and tumour infiltrating lymphocytes. *Int. J. Cancer*, 27, 265.

NAIRN, R. C. (1976) Immunological reactions in human cancer. III. Carcinoma of the colon and squamous cell carcinoma of the skin. In *Scientific Foundations of Oncology* (Ed. Symington & Carter). London: Heinemann. p. 549.

NIND, A. P. P., NAIRN, R. C., ROLLAND, J. M., GULI, E. P. G. & HUGHES, E. S. R. (1973) Lymphocyte anergy in patients with carcinoma. *Br. J. Cancer*, **28**, 108.

ROBINS, R. A., FLANNERY, G. R. & BALDWIN, R. W. (1979) Tumour-derived lymphoid cells prevent tumour growth in Winn assays. *Br. J. Cancer*, **40**, 946.

SVENNEVIG, J. S. (1980) T lymphocytes in malignant non-lymphoid human tumours detected by esterase technique. *Scand. J. Immunol.*, **12**, 513.

TOTTERMAN, T. H., HAYRY, P., SAKSELA, E., TIMONEN, T. & EKLUND, B. (1978) Cytological and functional analysis of inflammatory infiltrates in human malignant tumours. II. Functional investigations of the infiltrating inflammatory cells. *Eur. J. Immunol.*, **8**, 782.

UNDERWOOD, J. C. E. (1974) Lymphoreticular-cell infiltration in human tumours, prognostic and histological implications. A review. *Br. J. Cancer*, **30**, 538.

VANKY, F. T., ARGOV, S. A., EINHORN, S. A. & KLEIN, E. (1980) Role of alloantigens in natural killing. *J. Exp. Med.*, **151**, 1151.

VOSE, B. M., MOORE, M., GALLAGHER, P. & SCHOFIELD, P. F. (1980) Auto-reactive lymphocytes in colonic carcinoma. *Br. J. Cancer*, **42**, 177.

VOSE, B. M., VANKY, F. & KLEIN, E. (1977) Lymphocyte cytotoxicity against autologous tumour biopsy cells in humans. *Int. J. Cancer*, **20**, 512.

WERKMEISTER, J. A., PIHL, E., NIND, A. A. P., FLANNERY, G. R. & NAIRN, R. C. (1979) Immunoreactivity by intrinsic lymphoid cells in colorectal carcinoma. *Br. J. Cancer*, **40**, 839.

ZINKERNAGEL, R. M. & DOHERTY, P. C. (1974) Restriction of *in vitro* T cell-mediated cytotoxicity in lymphocytic choriomeningitis within a syngeneic or semi-allogeneic system. *Nature*, **248**, 701.